# 4,4′-Di-*tert*-butyl-2,2′-bipyridine

**DOI:** 10.1107/S1600536809029109

**Published:** 2009-07-31

**Authors:** Tatiana R. Amarante, Sónia Figueiredo, André D. Lopes, Isabel S. Gonçalves, Filipe A. Almeida Paz

**Affiliations:** a Department of Chemistry, University of Aveiro, CICECO, 3810-193 Aveiro, Portugal; b Faculty of Science and Technology, CIQA, University of the Algarve, Campus de Gambelas, 8005-139 Faro, Portugal

## Abstract

In the title compound, C_18_H_24_N_2_, the mol­ecular unit adopts a *trans* conformation around the central C—C bond [N—C—C—N torsion angle of 179.2 (3)°], with the two aromatic rings almost coplanar [dihedral angle of only 0.70 (4)°]. The crystal packing is driven by co-operative contacts involving weak C—H⋯N and C—H⋯π inter­actions, and also the need to fill effectively the available space.

## Related literature

For related structures, see: Batsanov *et al.* (2007 ▶); Coelho *et al.* (2007 ▶); Paz & Klinowski (2003 ▶); Paz *et al.* (2002 ▶). For a description of the Cambridge Structural Database, see: Allen (2002 ▶).
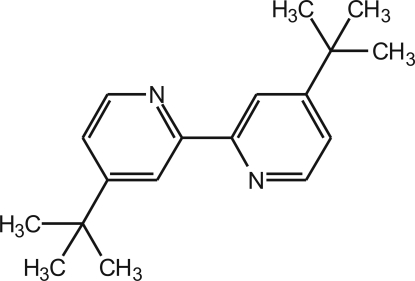


## Experimental

### 

#### Crystal data

C_18_H_24_N_2_
*M* *_r_* = 268.39
Monoclinic, 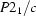

*a* = 10.241 (5) Å
*b* = 6.228 (3) Å
*c* = 24.559 (10) Å
β = 99.75 (3)°
*V* = 1543.7 (12) Å^3^
*Z* = 4
Mo *K*α radiation
μ = 0.07 mm^−1^
*T* = 296 K
0.20 × 0.16 × 0.14 mm

#### Data collection

Bruker X8 Kappa CCD APEXII diffractometer
Absorption correction: multi-scan (*SADABS*; Sheldrick, 1997 ▶) *T*_min_ = 0.98, *T*_max_ = 0.99
15295 measured reflections
2722 independent reflections
1805 reflections with *I* > 2σ(*I*)
*R*_int_ = 0.044

#### Refinement

*R*[*F* ^2^ > 2σ(*F* ^2^)] = 0.080
*wR*(*F* ^2^) = 0.217
*S* = 1.12
2722 reflections
187 parameters
H-atom parameters constrained
Δρ_max_ = 0.29 e Å^−3^
Δρ_min_ = −0.44 e Å^−3^

### 

Data collection: *APEX2* (Bruker, 2006 ▶); cell refinement: *SAINT-Plus* (Bruker, 2005 ▶); data reduction: *SAINT-Plus*; program(s) used to solve structure: *SHELXTL* (Sheldrick, 2008 ▶); program(s) used to refine structure: *SHELXTL*; molecular graphics: *DIAMOND* (Brandenburg, 2009 ▶); software used to prepare material for publication: *SHELXTL*.

## Supplementary Material

Crystal structure: contains datablocks global, I. DOI: 10.1107/S1600536809029109/bg2282sup1.cif

Structure factors: contains datablocks I. DOI: 10.1107/S1600536809029109/bg2282Isup2.hkl

Additional supplementary materials:  crystallographic information; 3D view; checkCIF report

**Fig. 3.**
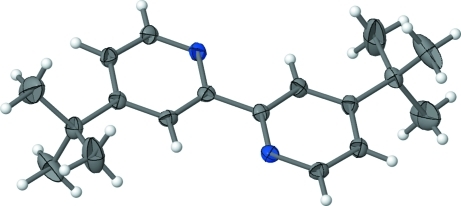
Asymmetric unit of the title compound with all non-hydrogen atoms represented as thermal ellipsoids drawn at the 50% probability level.

Enhanced figure: interactive version of Fig. 3

**Fig. 4.**
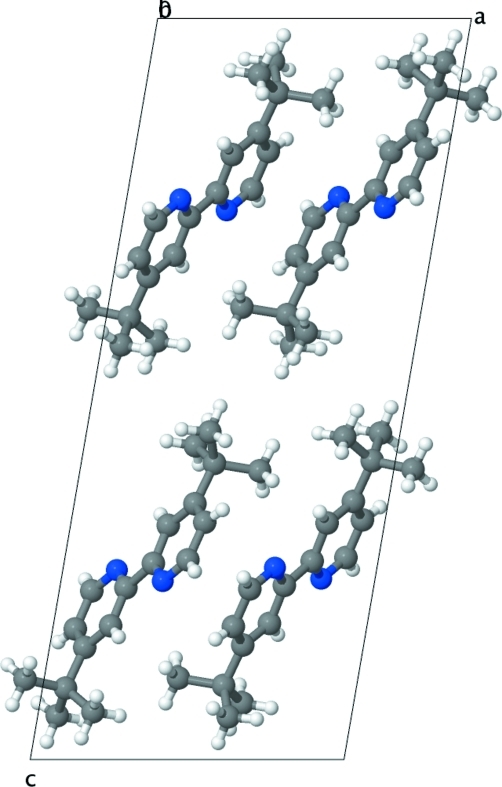
Crystal packing of the title compound viewed along the [010] direction of the unit cell.

Enhanced figure: interactive version of Fig. 4

## Figures and Tables

**Table 1:** Hydrogen-bond geometry (Å, °)

*D*—H⋯*A*	*D*—H	H⋯*A*	*D*⋯*A*	*D*—H⋯*A*
C12—H12*B*⋯N1^i^		2.74	3.637 (4)	155
C12—H12*A*⋯*Cg*2^ii^			3.78	140
C1—H1⋯*Cg*1^ii^			3.40	137

Symmetry codes: (i) 
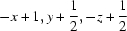
; (ii) 
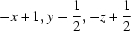
. *Cg*1 and *Cg*2 are the centroids of the N1,C1–C5 and N2,C6–C10 rings, respectively.

**Fig. 1.**
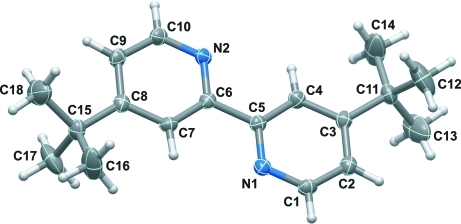
Schematic representation of the molecular unit of the title compound, with non-hydrogen atoms being represented as thermal displacement ellipsoids drawn at the 50% probability level. The atomic labeling is provided for all non-hydrogen atoms. Bond lengths and angles are provided as supplementary material.

**Fig. 2.**
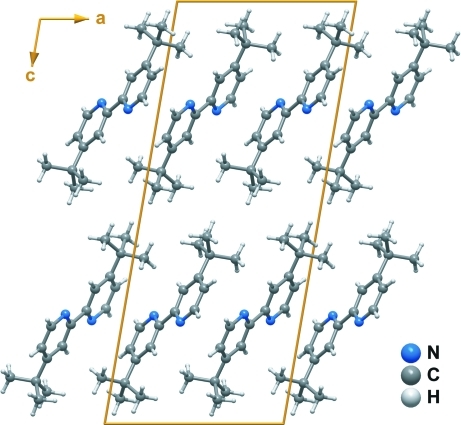
Crystal packing of the title compound viewed along the b axis of the unit cell.

## supplementary crystallographic information

### Comment

Organic derivatives of 2,2'-bipyridine find innumerous applications in the field of synthetic chemistry, in particular as *N*,*N*-chelating agents which are able to coordinate to a myriad of metal centres. A search in the literature and in the Cambridge Structural Database (CSD, Version of November 2008 with three updates; Allen, 2002) reveals that the title compound has been predominantely employed in the coordination chemistry field with only the structure by Batsanov *et al.* (2007) being of an organic crystal in which the title compound co-crystallizes with hexafluorobenzene: C_18_H_24_N_2_^.^C_6_F_6_. Following our interest on organic crystals with pyridine derivatives (Coelho *et al.*, 2007; Paz & Klinowski, 2003; Paz *et al.*, 2002) we wish to report the structure of the title compound (I) at 150K.

The asymmetric unit is composed of an entire molecular unit as depicted in Fig. 1. The molecule adopts in the crystal structure a *trans* conformation around the central C—C bond, a feature also reported by Batsanov *et al.* for the co-crystal with hexafluorobenzene. This conformation seems to minimize steric repulsion between the substituent *tert*-butyl groups and the heteroatoms from the aromatic rings. While in the structure of Batsanov *et al.* the 4,4'-di-*tert*-butyl-2,2'-dipyridyl residue is structurally located on a mirror plane, which ensures coplanarity for the two aromatic rings, in the standalone crystal here reported the atoms are located on generic positions. Nevertheless, the average planes containing the two aromatic rings subtend a dihedral angle of only *ca* 0.70°, with the corresponding <(N1—C5—C6—N2) torsion angle around the central bond being of 179.2 (3)°.

Individual molecules close pack in the solid state forming layers placed in the (001) plane of the unit cell (Fig. 2). The presence of the large *tert*-butyl groups seems to prevent the presence of π-π stacking interactions as it can be easily observed by manipulating Enhanced Fig. 4. We note the existence of a terminal —CH_3_ group engaged in a C—H···N hydrogen bonding interaction: even though this contact is considered as weak (*d*_D···A_ being *ca* 3.64 Å) it is directional with <(DHA) being above 150° (Table 1). In addition, the same —CH_3_ group is involved in a C—H···π contact with the aromatic ring of an adjacent molecular unit [not shown; *d*_C···__π_ = *ca* 3.78 Å; <(C12—H12A···π) = *ca* 140°]. A similar contact connects two adjacent aromatic rings [not shown; *d*_C1···__π_ = *ca* 3.40 Å; <(C1—H1···π) = *ca* 137°]. Besides these weak cooperative interactions, close packing in (I) is further mediated by van der Waals interactions so to promote an effective filling of the available space. Noteworthy, in the C_18_H_24_N_2_^.^C_6_F_6_ organic crystal π-π contacts mediate the close packing because the auxiliary C_6_F_6_ molecule is small and can easily be accommodated on top of the 2,2'-dipyridyl residue.

### Experimental

4,4'-Di-*tert*-butyl-2,2'-dipyridyl was purchased from Sigma-Aldrich (98% purity) and used as received without further purification. Single crystals were isolated from the slow evaporation (at ambient temperature) over the period of one month from a solution of the title compound in toluene (Sigma-Aldrich, ACS reagent, >99.5%).

### Refinement

Hydrogen atoms bound to carbon were located at their idealized positions and were included in the final structural model in riding-motion approximation with C—H = 0.93 (aromatic C—H) or 0.96 Å (for the —CH_3_ moieties). The isotropic thermal displacement parameters for these atoms were fixed at 1.2 or 1.5 for the aromatic C—H or the —CH_3_ moieties, respectively, times *U*_eq_(C).

### Crystal data

**Table:**

C_18_H_24_N_2_	*F*(000) = 584
*M**_r_* = 268.39	*D*_x_ = 1.155 Mg m^−^^3^
Monoclinic, *P*2_1_/*c*	Mo *K*α radiation, λ = 0.71073 Å
Hall symbol: -P 2ybc	Cell parameters from 5252 reflections
*a* = 10.241 (5) Å	θ = 3.4–25.3°
*b* = 6.228 (3) Å	µ = 0.07 mm^−^^1^
*c* = 24.559 (10) Å	*T* = 296 K
β = 99.75 (3)°	Plate, colourless
*V* = 1543.7 (12) Å^3^	0.20 × 0.16 × 0.14 mm
*Z* = 4	

### Data collection

**Table:**

Bruker X8 Kappa CCD APEXII diffractometer	2722 independent reflections
Radiation source: fine-focus sealed tube	1805 reflections with *I* > 2σ(*I*)
graphite	*R*_int_ = 0.044
ω and φ scans	θ_max_ = 25.3°, θ_min_ = 3.6°
Absorption correction: multi-scan (*SADABS*; Sheldrick, 1997)	*h* = −12→11
*T*_min_ = 0.98, *T*_max_ = 0.99	*k* = −7→5
15295 measured reflections	*l* = −29→29

### Refinement

**Table:**

Refinement on *F*^2^	Primary atom site location: structure-invariant direct methods
Least-squares matrix: full	Secondary atom site location: difference Fourier map
*R*[*F*^2^ > 2σ(*F*^2^)] = 0.080	Hydrogen site location: inferred from neighbouring sites
*wR*(*F*^2^) = 0.217	H-atom parameters constrained
*S* = 1.12	*w* = 1/[σ^2^(*F*_o_^2^) + (0.0449*P*)^2^ + 4.7425*P*] where *P* = (*F*_o_^2^ + 2*F*_c_^2^)/3
2722 reflections	(Δ/σ)_max_ < 0.001
187 parameters	Δρ_max_ = 0.29 e Å^−^^3^
0 restraints	Δρ_min_ = −0.44 e Å^−^^3^

### Special details

**Table:**

Geometry. All e.s.d.'s (except the e.s.d. in the dihedral angle between two l.s. planes) are estimated using the full covariance matrix. The cell e.s.d.'s are taken into account individually in the estimation of e.s.d.'s in distances, angles and torsion angles; correlations between e.s.d.'s in cell parameters are only used when they are defined by crystal symmetry. An approximate (isotropic) treatment of cell e.s.d.'s is used for estimating e.s.d.'s involving l.s. planes.
Refinement. Refinement of *F*^2^ against ALL reflections. The weighted *R*-factor *wR* and goodness of fit *S* are based on *F*^2^, conventional *R*-factors *R* are based on *F*, with *F* set to zero for negative *F*^2^. The threshold expression of *F*^2^ > σ(*F*^2^) is used only for calculating *R*-factors(gt) *etc*. and is not relevant to the choice of reflections for refinement. *R*-factors based on *F*^2^ are statistically about twice as large as those based on *F*, and *R*- factors based on ALL data will be even larger.

### Fractional atomic coordinates and isotropic or equivalent isotropic displacement parameters (Å^2^)

**Table:**

	*x*	*y*	*z*	*U*_iso_*/*U*_eq_	
N1	0.3249 (3)	0.3001 (5)	0.25918 (12)	0.0246 (7)	
N2	0.1719 (3)	0.8151 (5)	0.24419 (12)	0.0243 (7)	
C1	0.3931 (3)	0.1715 (6)	0.23049 (14)	0.0252 (8)	
H1	0.4201	0.0386	0.2456	0.030*	
C2	0.4260 (3)	0.2236 (6)	0.17986 (14)	0.0256 (9)	
H2	0.4757	0.1284	0.1625	0.031*	
C3	0.3852 (3)	0.4166 (6)	0.15491 (13)	0.0227 (8)	
C4	0.3118 (3)	0.5477 (6)	0.18389 (14)	0.0240 (8)	
H4	0.2801	0.6786	0.1689	0.029*	
C5	0.2852 (3)	0.4848 (6)	0.23540 (14)	0.0218 (8)	
C6	0.2103 (3)	0.6285 (6)	0.26780 (14)	0.0213 (8)	
C7	0.1830 (3)	0.5671 (6)	0.31914 (14)	0.0226 (8)	
H7	0.2130	0.4351	0.3340	0.027*	
C8	0.1115 (3)	0.7010 (6)	0.34851 (14)	0.0233 (8)	
C9	0.0721 (3)	0.8944 (6)	0.32347 (14)	0.0259 (9)	
H9	0.0241	0.9917	0.3409	0.031*	
C10	0.1041 (3)	0.9434 (6)	0.27250 (15)	0.0258 (9)	
H10	0.0763	1.0752	0.2569	0.031*	
C11	0.4250 (4)	0.4806 (7)	0.10003 (14)	0.0272 (9)	
C12	0.5727 (4)	0.5224 (11)	0.11030 (19)	0.0645 (17)	
H12A	0.6188	0.3938	0.1238	0.097*	
H12B	0.5929	0.6345	0.1372	0.097*	
H12C	0.6000	0.5655	0.0764	0.097*	
C13	0.3929 (6)	0.3050 (10)	0.05788 (19)	0.0722 (19)	
H13A	0.4108	0.3540	0.0228	0.108*	
H13B	0.3009	0.2675	0.0545	0.108*	
H13C	0.4464	0.1813	0.0694	0.108*	
C14	0.3543 (6)	0.6789 (11)	0.0762 (2)	0.075 (2)	
H14A	0.3818	0.7133	0.0417	0.112*	
H14B	0.3754	0.7963	0.1015	0.112*	
H14C	0.2604	0.6542	0.0701	0.112*	
C15	0.0734 (4)	0.6350 (7)	0.40367 (14)	0.0277 (9)	
C16	0.1739 (5)	0.4837 (10)	0.43529 (18)	0.0592 (16)	
H16A	0.1497	0.4522	0.4705	0.089*	
H16B	0.2599	0.5496	0.4407	0.089*	
H16C	0.1761	0.3530	0.4147	0.089*	
C17	−0.0606 (5)	0.5247 (10)	0.39153 (18)	0.0558 (15)	
H17A	−0.1247	0.6214	0.3717	0.084*	
H17B	−0.0877	0.4840	0.4256	0.084*	
H17C	−0.0541	0.3989	0.3695	0.084*	
C18	0.0621 (6)	0.8287 (9)	0.44040 (18)	0.0594 (15)	
H18A	−0.0077	0.9208	0.4229	0.089*	
H18B	0.1443	0.9062	0.4462	0.089*	
H18C	0.0426	0.7812	0.4753	0.089*	

### Atomic displacement parameters (Å^2^)

**Table:**

	*U*^11^	*U*^22^	*U*^33^	*U*^12^	*U*^13^	*U*^23^
N1	0.0221 (16)	0.0236 (19)	0.0285 (16)	−0.0027 (14)	0.0056 (12)	−0.0015 (13)
N2	0.0191 (15)	0.0244 (19)	0.0306 (16)	0.0026 (14)	0.0073 (12)	0.0036 (14)
C1	0.0225 (19)	0.021 (2)	0.0313 (19)	0.0014 (16)	0.0021 (15)	0.0012 (16)
C2	0.0210 (19)	0.029 (2)	0.0274 (18)	−0.0026 (17)	0.0044 (15)	−0.0060 (16)
C3	0.0175 (18)	0.027 (2)	0.0240 (18)	−0.0001 (16)	0.0029 (14)	−0.0027 (16)
C4	0.0197 (18)	0.026 (2)	0.0256 (18)	0.0016 (16)	0.0027 (14)	0.0020 (16)
C5	0.0166 (17)	0.023 (2)	0.0249 (17)	0.0002 (16)	0.0023 (14)	−0.0001 (15)
C6	0.0165 (17)	0.021 (2)	0.0256 (18)	−0.0033 (15)	0.0013 (14)	−0.0043 (15)
C7	0.0215 (18)	0.019 (2)	0.0260 (18)	0.0003 (16)	0.0015 (14)	0.0009 (15)
C8	0.0178 (18)	0.026 (2)	0.0263 (18)	−0.0042 (16)	0.0031 (14)	−0.0051 (16)
C9	0.0205 (19)	0.026 (2)	0.0320 (19)	0.0022 (16)	0.0064 (15)	−0.0027 (16)
C10	0.0198 (18)	0.024 (2)	0.0336 (19)	0.0025 (16)	0.0055 (15)	0.0023 (17)
C11	0.026 (2)	0.033 (2)	0.0240 (18)	−0.0017 (17)	0.0078 (15)	0.0013 (16)
C12	0.041 (3)	0.112 (5)	0.043 (3)	−0.022 (3)	0.012 (2)	0.017 (3)
C13	0.118 (5)	0.071 (4)	0.034 (3)	−0.030 (4)	0.031 (3)	−0.013 (3)
C14	0.102 (5)	0.085 (5)	0.049 (3)	0.046 (4)	0.045 (3)	0.034 (3)
C15	0.028 (2)	0.033 (2)	0.0227 (18)	0.0033 (18)	0.0056 (15)	−0.0009 (16)
C16	0.061 (3)	0.085 (4)	0.036 (2)	0.028 (3)	0.021 (2)	0.025 (3)
C17	0.050 (3)	0.084 (4)	0.037 (2)	−0.031 (3)	0.018 (2)	−0.003 (3)
C18	0.100 (4)	0.048 (3)	0.036 (2)	0.001 (3)	0.028 (3)	−0.002 (2)

### Geometric parameters (Å, °)

**Table:**

N1—C5	1.323 (5)	C11—C12	1.513 (6)
N1—C1	1.338 (5)	C12—H12A	0.9600
N2—C6	1.328 (5)	C12—H12B	0.9600
N2—C10	1.329 (5)	C12—H12C	0.9600
C1—C2	1.381 (5)	C13—H13A	0.9600
C1—H1	0.9300	C13—H13B	0.9600
C2—C3	1.382 (5)	C13—H13C	0.9600
C2—H2	0.9300	C14—H14A	0.9600
C3—C4	1.385 (5)	C14—H14B	0.9600
C3—C11	1.526 (5)	C14—H14C	0.9600
C4—C5	1.395 (5)	C15—C16	1.510 (6)
C4—H4	0.9300	C15—C17	1.518 (6)
C5—C6	1.493 (5)	C15—C18	1.522 (6)
C6—C7	1.390 (5)	C16—H16A	0.9600
C7—C8	1.389 (5)	C16—H16B	0.9600
C7—H7	0.9300	C16—H16C	0.9600
C8—C9	1.382 (5)	C17—H17A	0.9600
C8—C15	1.529 (5)	C17—H17B	0.9600
C9—C10	1.381 (5)	C17—H17C	0.9600
C9—H9	0.9300	C18—H18A	0.9600
C10—H10	0.9300	C18—H18B	0.9600
C11—C14	1.500 (6)	C18—H18C	0.9600
C11—C13	1.503 (6)		
			
C5—N1—C1	115.9 (3)	H12A—C12—H12B	109.5
C6—N2—C10	116.1 (3)	C11—C12—H12C	109.5
N1—C1—C2	124.2 (4)	H12A—C12—H12C	109.5
N1—C1—H1	117.9	H12B—C12—H12C	109.5
C2—C1—H1	117.9	C11—C13—H13A	109.5
C1—C2—C3	120.1 (3)	C11—C13—H13B	109.5
C1—C2—H2	119.9	H13A—C13—H13B	109.5
C3—C2—H2	119.9	C11—C13—H13C	109.5
C2—C3—C4	115.8 (3)	H13A—C13—H13C	109.5
C2—C3—C11	120.9 (3)	H13B—C13—H13C	109.5
C4—C3—C11	123.3 (3)	C11—C14—H14A	109.5
C3—C4—C5	120.4 (4)	C11—C14—H14B	109.5
C3—C4—H4	119.8	H14A—C14—H14B	109.5
C5—C4—H4	119.8	C11—C14—H14C	109.5
N1—C5—C4	123.5 (3)	H14A—C14—H14C	109.5
N1—C5—C6	115.7 (3)	H14B—C14—H14C	109.5
C4—C5—C6	120.8 (3)	C16—C15—C17	109.5 (4)
N2—C6—C7	123.1 (3)	C16—C15—C18	107.7 (4)
N2—C6—C5	115.6 (3)	C17—C15—C18	108.6 (4)
C7—C6—C5	121.3 (3)	C16—C15—C8	111.7 (3)
C8—C7—C6	120.7 (4)	C17—C15—C8	107.8 (3)
C8—C7—H7	119.7	C18—C15—C8	111.6 (4)
C6—C7—H7	119.7	C15—C16—H16A	109.5
C9—C8—C7	115.6 (3)	C15—C16—H16B	109.5
C9—C8—C15	122.0 (3)	H16A—C16—H16B	109.5
C7—C8—C15	122.4 (3)	C15—C16—H16C	109.5
C10—C9—C8	120.0 (3)	H16A—C16—H16C	109.5
C10—C9—H9	120.0	H16B—C16—H16C	109.5
C8—C9—H9	120.0	C15—C17—H17A	109.5
N2—C10—C9	124.5 (4)	C15—C17—H17B	109.5
N2—C10—H10	117.7	H17A—C17—H17B	109.5
C9—C10—H10	117.7	C15—C17—H17C	109.5
C14—C11—C13	107.2 (4)	H17A—C17—H17C	109.5
C14—C11—C12	109.0 (4)	H17B—C17—H17C	109.5
C13—C11—C12	109.6 (4)	C15—C18—H18A	109.5
C14—C11—C3	112.1 (3)	C15—C18—H18B	109.5
C13—C11—C3	111.0 (3)	H18A—C18—H18B	109.5
C12—C11—C3	107.9 (3)	C15—C18—H18C	109.5
C11—C12—H12A	109.5	H18A—C18—H18C	109.5
C11—C12—H12B	109.5	H18B—C18—H18C	109.5
			
C5—N1—C1—C2	1.9 (5)	C6—C7—C8—C9	−0.5 (5)
N1—C1—C2—C3	−1.7 (6)	C6—C7—C8—C15	176.8 (3)
C1—C2—C3—C4	0.1 (5)	C7—C8—C9—C10	0.1 (5)
C1—C2—C3—C11	177.7 (3)	C15—C8—C9—C10	−177.2 (3)
C2—C3—C4—C5	1.2 (5)	C6—N2—C10—C9	−0.2 (5)
C11—C3—C4—C5	−176.3 (3)	C8—C9—C10—N2	0.2 (6)
C1—N1—C5—C4	−0.5 (5)	C2—C3—C11—C14	171.5 (4)
C1—N1—C5—C6	−179.4 (3)	C4—C3—C11—C14	−11.1 (6)
C3—C4—C5—N1	−1.1 (5)	C2—C3—C11—C13	51.7 (5)
C3—C4—C5—C6	177.8 (3)	C4—C3—C11—C13	−131.0 (4)
C10—N2—C6—C7	−0.3 (5)	C2—C3—C11—C12	−68.5 (5)
C10—N2—C6—C5	179.4 (3)	C4—C3—C11—C12	108.9 (5)
N1—C5—C6—N2	179.2 (3)	C9—C8—C15—C16	−152.6 (4)
C4—C5—C6—N2	0.2 (5)	C7—C8—C15—C16	30.2 (5)
N1—C5—C6—C7	−1.1 (5)	C9—C8—C15—C17	87.1 (5)
C4—C5—C6—C7	179.9 (3)	C7—C8—C15—C17	−90.1 (4)
N2—C6—C7—C8	0.6 (5)	C9—C8—C15—C18	−32.0 (5)
C5—C6—C7—C8	−179.0 (3)	C7—C8—C15—C18	150.8 (4)

### Hydrogen-bond geometry (Å, °)

**Table:**

*D*—H···*A*	*D*—H	H···*A*	*D*···*A*	*D*—H···*A*
C12—H12B···N1^i^	.	2.74	3.637 (4)	155
C12—H12A···Cg2^ii^	.	.	3.78	140
C1—H1···Cg1^ii^	.	.	3.40	137

Symmetry codes: (i) −*x*+1, *y*+1/2, −*z*+1/2; (ii) −*x*+1, *y*−1/2, −*z*+1/2.
